# Quantile regression for count data: jittering versus regression coefficients modelling in the analysis of credits earned by university students after remote teaching

**DOI:** 10.1007/s10260-022-00661-2

**Published:** 2022-10-12

**Authors:** Viviana Carcaiso, Leonardo Grilli

**Affiliations:** 1 Department of Statistical Sciences, University of Padua, Padova, Italy; 2 Department of Statistics, Computer Science and Applications ‘G. Parenti’, University of Florence, Firenze, Italy

**Keywords:** COVID-19, Integrated loss function, Quantile regression coefficients modelling (QRCM), R package qrcm, Remote teaching, University credits

## Abstract

The extension of quantile regression to count data raises several issues. We compare the traditional approach, based on transforming the count variable using jittering, with a recently proposed approach in which the coefficients of quantile regression are modelled by parametric functions. We exploit both methods to analyse university students’ data to evaluate the effect of emergency remote teaching due to COVID-19 on the number of credits earned by the students. The coefficients modelling approach performs a smoothing that is especially convenient in the tails of the distribution, preventing abrupt changes in the point estimates and increasing precision. Nonetheless, model selection is challenging because of the wide range of options and the limited availability of diagnostic tools. Thus the jittering approach remains fundamental to guide the choice of the parametric functions.

## Introduction

The statistical modelling of students’ productivity at university is challenging due to the nature of the response variable. Indeed, the number of credits earned by a student is a count variable with an irregular distribution since exams yield different credits and the sequence of exams varies across students. In this paper, we rely on quantile regression (Koenker and Bassett 1978; Koenker 2005; Kneib 2013; Davino et al. 2014), which is a methodology to analyse the relationships between the quantiles of the response variable and a set of explanatory variables. It is a flexible approach as it does not require specifying a parametric distribution for the response. In the traditional approach to quantile regression, the association between the response variable and the covariates is defined by a set of quantile-specific regression equations which are fitted separately, allowing to assess the effect of the covariates at each quantile of interest.

Quantile regression has been successfully applied to the evaluation of the performance of university students, focusing on continuous outcomes (Birch and Miller 2006; Adelfio et al. 2014). However, analysing a count variable such as the number of gained credits is challenging since most of the theoretical developments of quantile regression concern continuous outcomes and the extension to count data raises several issues. The main problem stems from the conjunction of a non-differentiable sample objective function with a discrete response variable. Such difficulties can be overcome by the jittering approach proposed by Machado and Santos Silva (2005): the idea is to add a uniform random variable on [0, 1) to the original count variable to obtain a continuous working variable whose quantiles have a one-to-one relationship with those of the count variable. This approach has been applied to different kinds of data, such as fertility data (Miranda 2008; Booth and Kee 2009), frequency of individual doctor visits (Winkelmann 2006; Moreira and Barros 2010), traffic accidents (Qin and Reyes 2011) and capacity of a pre-enrollment test to predict student performance (Grilli et al. 2016). Lee and Neocleous (2010) developed a Bayesian implementation to analyse respiratory hospital admissions.

Recently, Frumento and Salvati (2021) proposed to analyse count data by extending the quantile regression coefficients modelling (QRCM) framework of Frumento and Bottai (2016). This approach avoids jittering and employs a parametric model to describe the quantile regression coefficient functions. The imposed parametric structure is expected to give advantages in terms of smoothness, interpretation, computational time and efficiency of the estimators. Furthermore, the QRCM framework allows the joint estimation of multiple quantiles (e.g., Kadane and Tokdar 2012; Reich 2012; Reich and Smith 2013; Yang and Tokdar 2017; Das and Ghosal 2017; Fabrizi et al. 2020). The idea of simultaneous quantile regression has been advocated as a solution to quantile crossing (e.g., He 1997; Bondell et al. 2010; Liu and Wu 2011). The main challenge in applying the QRCM approach is the model selection because of the inherent complexity and the lack of well-established principles.

In this paper, we exploit the jittering approach of Machado and Santos Silva (2005) and the coefficients modelling approach of Frumento and Salvati (2021) to analyse the effect on university students’ productivity of the emergency remote teaching adopted in the spring of 2020 to face the COVID-19 outbreak. In Italy, remote teaching started on 5 March 2020, just a few days after the beginning of the second semester. The idea, similar to that of González et al. (2020), Meeter et al. (2020) and Iglesias-Pradas et al. (2021), is to compare the productivity of different cohorts of students. Specifically, we focus on the performance of first-year students during the second semester: the cohort 2019 experienced remote teaching, whereas the cohort 2018 attended standard face-to-face lessons. We conduct the analysis on first-year students of bachelor’s degrees in Psychology and Industrial Design at the University of Florence. These degree programs have been chosen because they have the same courses in the two years under comparison while being different in discipline and type of access (enrollment in Psychology is subject to an admission test, whereas it is free for Industrial Design).

We use data collected in the administrative archive on students’ careers, including information on passed exams and background characteristics, such as gender, high school type and grade. To evaluate the impact of remote teaching, we focus on the number of credits earned in the second semester, namely in the exam sessions between June and September. Specifically, we compare the credits earned by two cohorts: students enrolled in 2019 (treatment group) versus students enrolled in 2018 (control group). To adjust for possible differences in baseline productivity among the two cohorts, we condition on the number of credits obtained in the first semester, when both cohorts attended face-to-face lectures.

The rest of the paper is organised as follows. In Sect. 2 we outline the theory of two mentioned approaches to quantile regression for counts, namely jittering and parametric coefficients modelling. In Sect. 3 we illustrate the application of the two approaches for evaluating the effect of remote teaching on credits earned by the students of the degree program in Psychology. Finally, in Sect. 4 we discuss the main findings and compare the two approaches from a statistical modelling perspective. Appendix 1 reports the results for the degree program in Industrial Design.

## Methods

### Quantile regression

Standard regression methods aim at modelling the relationship between the conditional mean of the response variable and a set of covariates. Extended frameworks, such as GAMLSS (Stasinopoulos et al. 2018), consider a few higher order moments. On the other hand, quantile regression (Koenker and Bassett 1978; Koenker 2005; Kneib 2013; Davino et al. 2014) focuses on conditional quantiles, which fully describe the distribution of the response variable. Therefore, quantile regression avoids distributional assumptions, though it requires correctly specifying the regression function at all quantiles of interest. The main advantage of quantile regression lies in the possibility of investigating the effects of the covariates at different points of the distribution: for example, it may reveal that a covariate has a negligible effect at the 50$^{th}$ percentile (centre of the distribution) and a large effect at the 90$^{th}$ percentile (right tail).

We denote by $Y_{i}$ the response variable of interest for observation *i*, with $i=1,\dotsc ,n$; moreover, we denote by ${\varvec{x}}_{i}$ a $q+1$-dimensional vector with *q* observed covariates and a constant. The standard quantile regression (QR) model assumes that

1 $$\begin{aligned} Q_{T(Y_{i})}(p \vert {\varvec{x}}_{i}) = {\varvec{x}}_{i}'\varvec{\beta }(p), \end{aligned}$$

where *p* is the quantile order and $Q_{T(Y_{i})}(p \vert {\varvec{x}}_{i})$ is the conditional quantile function of a known, monotone transformation $T(\cdot )$ of $Y_{i}$. A transformed response may be convenient in the case of non-negative or bounded outcomes. The estimation of $\varvec{\beta }(p)$ at any fixed *p* is carried out by minimising the objective function

2 $$\begin{aligned} L(\varvec{\beta }(p)) = \sum _{i=1}^n (p-\omega _{p,i})(T(y_{i})-{\varvec{x}}_{i}' \varvec{\beta }(p)), \end{aligned}$$

where $y_{i}$ is a realisation of $Y_{i}$ and $\omega _{p,i} = I(T(y_{i}) \le {\varvec{x}}_{i}' \varvec{\beta }(p))$. In the rest of the paper, we simplify the notation by omitting the index *i*.

### Quantile regression for counts: the jittering approach

The conventional approach for analysing count data is to employ regression models for the conditional mean, that are typically based on the Poisson distribution and its generalisations. Quantile regression represents a flexible approach to analyse count data, even though most of the theoretical developments and empirical applications concern continuous outcomes. In fact, in linear quantile regression the response variable is assumed to be sampled from an absolutely continuous population, which is not true if *Y* is a count. The extension of quantile regression to count data raises several issues: when QR is applied to this kind of data a non-standard rate of convergence is obtained as a result of the non-smoothness of the objective function in combination with the discreteness of the response variable (Manski 1985). In addition, this may produce identifiability issues and computational problems (Frumento and Salvati 2021).

The solution suggested by Machado and Santos Silva (2005) is to construct a continuous random variable whose quantiles have a one-to-one relationship with the quantiles of the response variable. In particular, they propose to generate an artificial continuous variable *Z* by adding a uniform random variable *U* with support [0, 1) to the original count variable *Y*. This procedure, known as jittering (Stevens 1950), yields a working variable *Z* whose conditional quantile function is continuous in *p*. Thus, linear quantile regression can be applied to any monotone transformation $T(\cdot )$ of the working variable $Z = Y + U$:

3 $$\begin{aligned} Q_{T(Z)}(p\vert {\varvec{x}}) = {\varvec{x}}'\varvec{\beta }(p). \end{aligned}$$

Machado and Santos Silva (2005) propose using the logarithmic transformation, specifically

4 $$\begin{aligned} T(Z; p) = {\left\{ \begin{array}{ll} \log (Z-p) &{} \text {if } Z > p\\ \log (\zeta ) &{} \text {if } Z \le p \end{array}\right. } \end{aligned}$$

where $\zeta$ is a suitably small positive number introduced to guarantee that the transformation is feasible also for $Z \le p$. Given the estimate $\hat{\varvec{\beta }}(p)$, quantiles of the original count *Y* are consistently estimated by

5 $$\begin{aligned} {\hat{Q}}_{Y}(p\vert {\varvec{x}}) = \lceil T^{-1}({\varvec{x}}'\hat{\varvec{\beta }}(p)) - 1 \rceil , \end{aligned}$$

where $\lceil a \rceil$ denotes the ceiling function, which returns the smallest integer greater than or equal to *a*.

A drawback of the jittering approach is that the estimate $\hat{\varvec{\beta }}(p)$ depends not only on the data, but also on the specific realisation of the noise *U*. To attenuate this dependence, Machado and Santos Silva (2005) propose to repeat jittering and estimation *m* times and to average the resulting estimates. Denoting with $\hat{\varvec{\beta }}^{(l)}(p)$ the QR estimator based on the $l^{th}$ “jittered” sample, the “average jittering” estimator is

6 $$\begin{aligned} \hat{\varvec{\beta }}_{m} ^{A}(p) = \frac{1}{m} \sum _{l=1}^{m}\hat{\varvec{\beta }}^{(l)}(p). \end{aligned}$$

The “average jittering” estimator (6) is more efficient than the estimator obtained with a single sample. Moreover, it is consistent and asymptotically Normal. The simulation study of Machado and Santos Silva (2005) shows it has good properties in finite samples of size *n* = 500.

### Quantile regression for counts: the coefficients modelling approach

Recently, Frumento and Salvati (2021) proposed a promising approach to quantile regression for counts based on the framework of Quantile Regression Coefficients Modelling (QRCM). This framework, introduced by Frumento and Bottai (2016) for a continuous response, consists in linking the regression coefficients at different quantiles through suitable functions and jointly estimating the parameters by minimising an overall objective function. The QRCM paradigm has been applied to censored and truncated data (Frumento and Bottai 2017) and to longitudinal data (Frumento et al. 2021). The idea of Frumento and Salvati (2021) is to apply QRCM also to a discrete response such as a count, thus avoiding jittering. The QRCM approach provides numerous advantages, including parsimony, efficiency and ease of interpretation.

The idea of Frumento and Bottai (2016) is to specify parametric models for the quantile regression coefficient functions. Specifically, they define the regression parameters $\varvec{\beta }(p)$ as a function of *p* that depends on a finite-dimensional parameter $\varvec{\theta }$, that is

7 $$\begin{aligned} \varvec{\beta }(p\vert \varvec{\theta }) = \varvec{\theta }{\varvec{b}}(p), \end{aligned}$$

where ${\varvec{b}}(p)=[b_{1}(p),\dotsc ,b_{k}(p)]'$ is a set of *k* known basis functions of *p* and $\varvec{\theta }$ is a $(q+1) \times k$ matrix with entries $\theta _{cj}$, where *q* is the number of covariates (the model always includes an intercept). The quantile regression coefficient of the $c^{th}$ covariate is

8 $$\begin{aligned} \beta _{c}(p\vert \varvec{\theta }) = \theta _{c1}b_{1}(p) + \dotsc + \theta _{ck}b_{k}(p), \end{aligned}$$

$c=0,\dotsc ,q$. Under the structure of Eq. (7), the conditional quantile function is

9 $$\begin{aligned} Q_{T(Y)}(p\vert {\varvec{x}}, \varvec{\theta }) = {\varvec{x}}'\varvec{\theta }{\varvec{b}}(p). \end{aligned}$$

To reduce model complexity, some entries of the matrix $\varvec{\theta }$ may be set to 0 to allow the regression coefficients to be specified by subsets of the basis functions ${\varvec{b}}(p)$.

Quantile regression coefficients modelling (QRCM) is implemented in the qrcm R package (Frumento 2021). An estimate of $\varvec{\theta }$ is obtained by minimising the integrated objective function

10 $$\begin{aligned} {\bar{L}}(\varvec{\theta })=\int _0^{1}L(\varvec{\beta }(p\vert \varvec{\theta }))\text {d}p \, , \end{aligned}$$

which is the integral, with respect to the quantile order, of the loss function (2) of linear quantile regression.

This estimation approach, referred to as *integrated loss minimisation*, allows to estimate the entire quantile process instead of estimating a discrete set of quantiles. The model can be specified for any non-decreasing transformation of the outcome variable, though a linear model is the most common choice because of the simplicity of interpretation (Frumento and Bottai 2016).

Unlike the standard loss function of quantile regression, the integrated loss function displayed in (10) is a smooth function of its arguments: this allows to use standard algorithms, like Newton-Raphson or gradient search (Bottai et al. 2015), to perform minimisation, and to employ the standard theory of M-estimation (e.g., Newey and McFadden 1994) to investigate the asymptotic properties. Consistency and asymptotic normality hold under mild conditions. An advantage of the parametric approach is that inference does not require using bootstrap or estimating the sparsity function.

Frumento and Bottai (2016) argue that, in many instances, the estimator based on the integrated loss function is expected to more efficient than the standard quantile regression estimator, which takes one quantile at a time. Indeed, joint estimation with a parametric structure is likely to yield some gain in efficiency. Evidence in this direction is provided by the simulation results of Frumento and Bottai (2016), but this is an open question.

Standard quantile regression can be seen as a non-parametric version of the QRCM estimator, thus it can be used to explore the relationships in the data to guide the selection of the QRCM model.

The QRCM approach is defined for a continuous response. However, Frumento and Salvati (2021) suggest using the QRCM approach also for a count variable in order to solve the well-known issues outlined in Sect. 2.2. The idea is to parametrically model the conditional quantile function as if the response variable was continuous. This entails using a working model which does not reflect the actual distribution of the data, but, in the same spirit of Efron (1992), allows to estimate a smooth quantile function by minimising a smooth loss function.

The proposal of Frumento and Salvati (2021) is driven by the empirical evidence that the estimators obtained by applying QRCM to the jittered response $Z=Y+U$ or directly to $Y^{\circ }=Y+E[U]$ are almost identical. Such result follows from the imposed parametric structure, which allows to smooth away the mass points in the empirical data distribution. Therefore, after assuming without loss of generality that $E[U]=0.5$, model (9) is applied to a transformation $T(\cdot )$ of $Y^{\circ }=Y+0.5$, that is

11 $$\begin{aligned} Q_{T(Y^{\circ })}(p\vert {\varvec{x}}, \varvec{\theta }) = {\varvec{x}}'\varvec{\beta }(p\vert \varvec{\theta }) \, . \end{aligned}$$

Given that $Y^{\circ }$ is discrete, Eq. (11) should not be viewed as a data generating model, but as a convenient approximation. Indeed, Frumento and Salvati (2021) argue that “the idea of fitting a continuous quantile function to a discrete outcome should be regarded as a computational expedient and can be seen as an implicit way of performing jittering”.

Estimation of the parameters in $\varvec{\theta }$ is carried out by minimising the integrated loss function of Eq. (10). Given the estimate $\hat{\varvec{\theta }}$, the quantiles of *Y* are estimated as

12 $$\begin{aligned} Q_{Y}(p\vert {\varvec{x}},\hat{\varvec{\theta }}) = \lceil T^{-1}({\varvec{x}}'{\varvec{\beta }}(p\vert \hat{\varvec{\theta }})) - 1 \rceil , \end{aligned}$$

where $\lceil \cdot \rceil$ is the ceiling operator.

As for the transformation $T(\cdot )$ of the working variable, Frumento and Salvati (2021) point out that the identity and the *log* are natural choices. They prefer the identity due to the ease of interpretation, also noting that a log-linear association can be approximated by a linear model in which the covariates have been suitably transformed. The drawback of the linear specification with count data is that it does not ensure non-negative predicted quantiles. Nevertheless, the linear specification may still be preferable if the analysis aims at discovering relationships rather than making predictions.

The simulation study of Frumento and Salvati (2021) show that, if the “true” model is fitted, QRCM estimators are much more efficient than the estimators based on jittering. The gain in efficiency reduces as the specification of QRCM becomes more flexible, although relevant efficiency gains are still observed in the tails where the data are sparser. This suggests that describing $\varvec{\beta }(p\vert \varvec{\theta })$ by a parsimonious model can substantially improve the inference, but overfitting tends to nullify the gain.

In the QRCM approach, model building is challenging since $\varvec{\beta }(p\vert \varvec{\theta })$ can be specified in many different ways. Frumento and Salvati (2021) suggest using the structure with basis functions defined in Eq. (7), namely $\varvec{\beta }(p\vert \varvec{\theta }) = \varvec{\theta }{\varvec{b}}(p)$. Then, the main issue is the choice of the basis functions ${\varvec{b}}(p)$. In practice, any set of functions such that ${\varvec{b}}(p)$ induces a well-defined quantile function for some $\varvec{\theta }$ can be utilised, including polynomials $(p, p^{2}, p^{3},\dotsc )$, splines, piecewise linear functions, roots $(p^{1/2}, (1-p)^{1/2}, p^{1/3}, (1-p)^{1/3},\dotsc )$, logarithms $(\log (p)$, $-\log (1-p))$, trigonometric functions $(\cos (\pi p), \sin (\pi p))$, quantile functions of known distribution (e.g., that of a Normal, Beta or Gamma distribution), and combinations of the above. In practice, it is likely that some of the quantile regression coefficient functions are well approximated by simple functions of *p*, like a linear function or even a constant.

To carry out model selection, restrictions on $\varvec{\theta }$ can be evaluated using Wald tests. The minimised value of the integrated loss function (10) can be used to monitor the fit of competing models, but it does not account for model complexity. Moreover, the model that provides the best fit from a list of candidates may actually not fit the data properly: for instance, some functions in ${\varvec{b}}(p)$ may be inadequate or the effects of the covariates may not be linear at some quantiles. Frumento and Bottai (2016) propose a goodness-of-fit test comparing $F(y_{i}\vert \, {\varvec{x}}_{i},\varvec{\theta })$ with a uniform distribution *U*(0, 1). Indeed, under the true model, $F(y_{i}\vert \, {\varvec{x}}_{i},\varvec{\theta })$ is uniformly distributed for every $i = 1, \ldots , n$. The distance can be measured by the Kolmogorov-Smirnoff or Cramér-von Mises statistics, and a Monte Carlo approach can be used to compute the *p*-values.

## Case study: effect of remote teaching on student productivity

### Data and preliminary analysis

At the beginning of March 2020, the Italian government, following the increase in the number of cases of COVID-19, decided to close all schools and universities, which then implemented various forms of remote teaching. We aim to evaluate the impact of remote teaching on the productivity of the students of a couple of degree programs at the University of Florence. We take data from the administrative records of first-year students who enrolled in the bachelor’s degree programs of Psychology and Industrial Design in the academic years 2018/2019 and 2019/2020. We consider two degree programs to have insights into different fields of study. The idea, similar to that of Meeter et al. (2020), is to compare the productivity of first-year students in the second semester of 2019/20 who attended remote teaching to that of first-year students in the second semester of 2018/19 who attended face-to-face lectures. In the academic years under comparison, the study plan remained the same.

We excluded the students who did not earn any credit in the first semester (212 out of 861 for Psychology and 36 out of 333 for Industrial design). In fact, those students have an irregular career since in the second semester mostly take exams of first semester courses, which were not affected by remote teaching. In addition, those students have low ability or low motivation since they are likely to be unproductive also in the second semester (the proportion of students with zero credits also in the second semester is 0.495 for Psychology and 0.833 for Industrial design).

The dataset for the analysis consists of 649 first-year students in Psychology and 297 in Industrial Design. The available characteristics of the students are summarised in Table 1, together with the average and standard deviation of the number of credits earned in each semester (the expected number is about 30 per semester). The two degree programs have students with different characteristics, which are similar in the two cohorts. Students of Psychology have a higher productivity, which is not surprising given that they have been selected by a closed-number admission test.

**Table 1:** Summary of background characteristics and obtained credits of first-year students by degree program and year of enrollment (2018 or 2019), University of Florence

	Psychology			Industrial design		
	2018	2019	Total	2018	2019	Total
Nr. observations	313	336	649	139	158	297
*Gender (%)*						
Female	76.7	82.1	79.5	72.7	62.0	67.0
Male	23.3	17.2	20.5	27.3	34.0	33.0
*Type of high school (%)*						
Scientific	37.7	32.7	35.1	32.4	25.3	28.6
Humanities	16.3	17.0	16.6	5.04	6.96	6.06
Language	7.35	7.14	7.24	2.88	3.80	3.37
Human sciences	24.6	19.9	22.2	12.2	9.49	10.8
Art school	1.28	2.98	2.16	22.3	29.1	25.9
Technical	9.27	15.2	12.3	19.4	18.4	18.9
Other	3.51	5.06	4.31	5.76	6.97	6.40
*High School grade*						
Average	82.1	80.3	81.17	76.9	78.3	77.63
SD	10.9	11.1	11.00	10.1	11.1	10.79
*Credits in the *1*st* *semester*						
Average	21.8	21.5	21.6	16.7	17.0	16.9
SD	6.65	6.63	6.64	7.46	7.64	7.54
*Credits in the* 2*nd* *semester*						
Average	30.1	28.2	29.1	19.8	22.1	21.0
SD	9.23	9.85	9.59	9.82	9.51	9.70

### Modelling

**Fig. 1:**
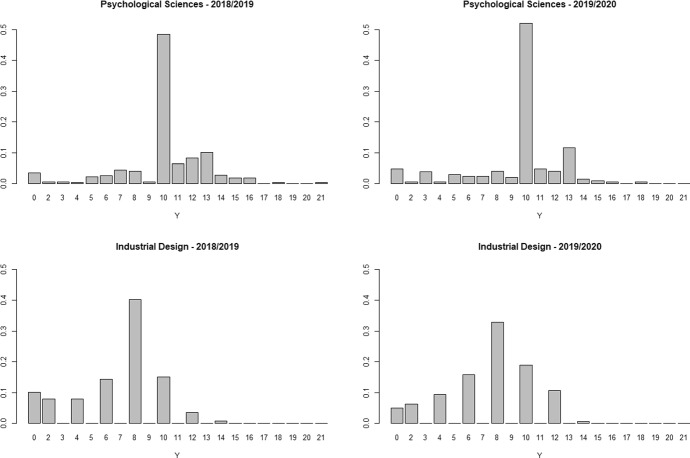
Distribution of credits obtained in the second semester for first-year students in Psychology and Industrial Design by academic year of enrollment (2018/2019 and 2019/2020), University of Florence

Students’ productivity is measured by the number of gained credits (ECTS). All considered exams provide 6, 9 or 12 credits. The number of credits to obtain, based on the study plan, is about 30 per semester. Students can achieve a higher number of credits than expected since, in the second semester, they can take exams they failed in the first one or exams of the following year. For each student, the number of credits earned in the first semester is the sum of the credits associated with the exams passed in January and February, whereas for the second semester the sum concerns the exams passed in June, July and September. We discard exams taken in other months and didactic activities without a grade. Given that the number of credits is always a multiple of 3, the response variable *Y* used in the models is defined as the number of credits earned in the second semester divided by 3. It follows that *Y* takes almost all integer values between 0 and 21. As shown in Fig. 1, the response variable has an irregular distribution in both degree programs since exams yield different credits and the sequence of exams varies across students. Even if the number of gained credits is not originated from a counting process, it has the support of a count variable. The lower bound is zero, whereas the upper bound is not fixed in advance since students can take extra exams. Also note that the sample distributions of Fig. 1 do not show any thickening of frequencies in the right tail. Therefore, the number of gained credits can be modelled as a count variable, and quantile regression is an appealing methodology to handle its uneven distribution. In a similar context, Grilli et al. (2016) applied quantile regression for counts based on jittering. Here we also apply the coefficients modelling approach (QRCM) and make a comparison.

The covariates to be included in the models are:

- $X_{1}$: number of credits obtained during the first semester, centred around its mean and scaled.
- $X_{2}$: dummy variable for cohort 2019 (remote teaching in the second semester) vs 2018 (face-to-face teaching in the second semester).
- $X_{3}$: dummy variable for male vs female.
- $X_{4}$: high school grade, centred around its mean and scaled.
- $X_{5},\dotsc ,X_{10}$: dummy variables for the type of high school (except for the baseline category “Scientific”).

The analysis is performed separately for each degree program. The effect of remote teaching on gained credits is summarised by the coefficient of the dummy variable $X_{2}$ for the cohort 2019. For the jittering approach we specify the following model:

13 $$\begin{aligned} Q_{T(Z,p)}(p\vert {\varvec{x}}) = {\varvec{x}}'\varvec{\beta }(p) = \beta _{0}(p) + \sum _{c=1}^{10} \beta _{c}(p)\,x_{c} , \end{aligned}$$

with $T(Z,p)=Z-p$. The subtraction of *p* is motivated by the fact that the quantile function of *Z* is bounded from below by *p*. Differently from Machado and Santos Silva (2005), who adopted the *log* transformation (4), here the transformation is linear. The corresponding model in the QRCM approach is

14 $$\begin{aligned} Q_{Y^{\circ }}(p\vert {\varvec{x}}, \varvec{\theta }) = {\varvec{x}}'\varvec{\beta }(p\vert \varvec{\theta }) = \beta _{0}(p\vert \varvec{\theta }) + \sum _{c=1}^{10} \beta _{c}(p\vert \varvec{\theta })\,x_{c} . \end{aligned}$$

where the coefficients are assumed to be linear combinations of basis functions as in Eq. (8), namely $\beta _{c}(p\vert \varvec{\theta }) = \theta _{c1}b_{1}(p) + \dotsc + \theta _{ck}b_{k}(p)$. Therefore, model specification entails choosing the basis functions and setting some parameters $\theta _{cj}$ at zero. Indeed, to avoid overfitting, for most regression coefficients it is reasonable to use a subset of the basis functions.

For the QRCM approach we specified a linear quantile regression model, corresponding to an identity function for the transformation of the working variable. To ensure comparability, we made the same choice for the jittering approach even if Machado and Santos Silva (2005) used the *log* transformation. As mentioned in Sect. 2.3, the linear specification in QRCM is advocated by Frumento and Salvati (2021) since it is easier to fit and interpret. Clearly, a linear model does not guarantee non-negative values of the count variable. To evaluate the impact of this issue in our application, we computed with Eq. (12) the predicted quantiles according to the selected QRCM specification for Psychology (see Model 2 of Table 2 further on). At $p=0.05$ we obtained $-1$ with frequency 14 and $-2$ with frequency 1. Overall, the negative values are small in magnitude and rare (15 out of 649, i.e. 2.3%), thus we proceed with the linear specification, also considering that we are going to exploit the model to infer relationships, rather than making predictions.

The jittering approach is implemented by the R package quantreg with jittering repeated 100 times (Koenker 2021), whereas the QRCM model is fitted using the iqr function of the qrcm package (Frumento 2021).

In the following, we report the results of the analysis concerning the program degree in Psychology, while the corresponding results for the program degree in Industrial Design are summarised in the Appendix.

### Results for Psychology

To implement the QRCM approach we fitted several models with various types of basis functions and different restrictions. Some of the fitted models are summarised in Table 2, showing the basis functions, the number of model parameters and the integrated loss function.

**Table 2:** Alternative QRCM specifications for the number of gained credits: basis functions for the quantile regression coefficients, number of parameters and minimised integrated loss function. Degree program in Psychology

Model	Intercept	$X_2$	$X_1, X_3 , X_4$	$X_5,\dotsc ,X_{10}$	Parameters	Loss
0	poly$(p,1)$	poly$(p,1)$	poly$(p,1)$	poly$(p,1)$	22	482.350
1	$\text {poly}(p,5),-\log (1-p)$	$\text {poly}(p,5)$	poly$(p,1)$	1	25	476.704
2	$\text {poly}(p,5),-\log (1-p)$	$\text {poly}(p,5),-\log (1-p)$	poly$(p,1)$	1	26	476.229
3	$\text {poly}(p,5),-\log (1-p)$	$\text {poly}(p,5),-\log (1-p)$	$\text {poly}(p,3)$	1	32	476.117
4	$\text {poly}(p,8),-\log (1-p)$	$\text {poly}(p,8),-\log (1-p)$	poly$(p,1)$	1	32	475.477

poly(*p*,*r*) denotes the shifted Legendre polynomials up to degree *r*; for example, poly(*p*,2) includes the terms $1, 2p-1, 6p^2-6p+1$ and gives the same fit as the standard polynomial $1, p, p^2$

Firstly, Table 2 reports a baseline model where all quantile regression coefficients are linear functions of *p* (Model 0). In the other models, the intercept $\beta _{0}(p\vert \varvec{\theta })$ and the coefficient for the effect of interest $\beta _{2}(p\vert \varvec{\theta })$ are modelled in a flexible way, whereas the other coefficients are approximated with low-order polynomials or even a constant. Models 1 and 2 only differ for the term $-log(1-p)$ in $\beta _{2}(p\vert \varvec{\theta })$. Models 3 and 4 are extensions of Model 2 with finer specification of some coefficients. We select Model 2, which is defined by the following equations:

$$\begin{aligned} \beta _{0}(p\vert \varvec{\theta })&= \theta _{00} + \theta _{01}p + \theta _{02}p^2 + \theta _{03}p^3+ \theta _{04}p^4+ \theta _{05}p^5 - \theta _{06}\log (1-p)\\ \beta _{2}(p\vert \varvec{\theta })&= \theta _{20}+ \theta _{21}p + \theta _{22}p^2 + \theta _{23}p^3+\theta _{24}p^4 + \theta _{25}p^5- \theta _{26}\log (1-p)\\ \beta _{c}(p\vert \varvec{\theta })&= \theta _{c0} + \theta _{c1}p \quad \text {for} \, c \in \{1,3,4\} \\ \beta _{c}(p\vert \varvec{\theta })&= \theta _{c0} \quad \text {for} \, c=5,6,\dotsc ,10, \end{aligned}$$

where the matrix $\varvec{\theta }$ has elements $\theta _{cj}$ with *c* denoting the quantile regression coefficient and *j* denoting the element of the basis function. The formula is written with standard polynomials for the sake of clarity. However, estimation is carried out with shifted Legendre polynomials, which are polynomials orthogonal on [0, 1] yielding the same fit, but improving computation.

**Fig. 2:**
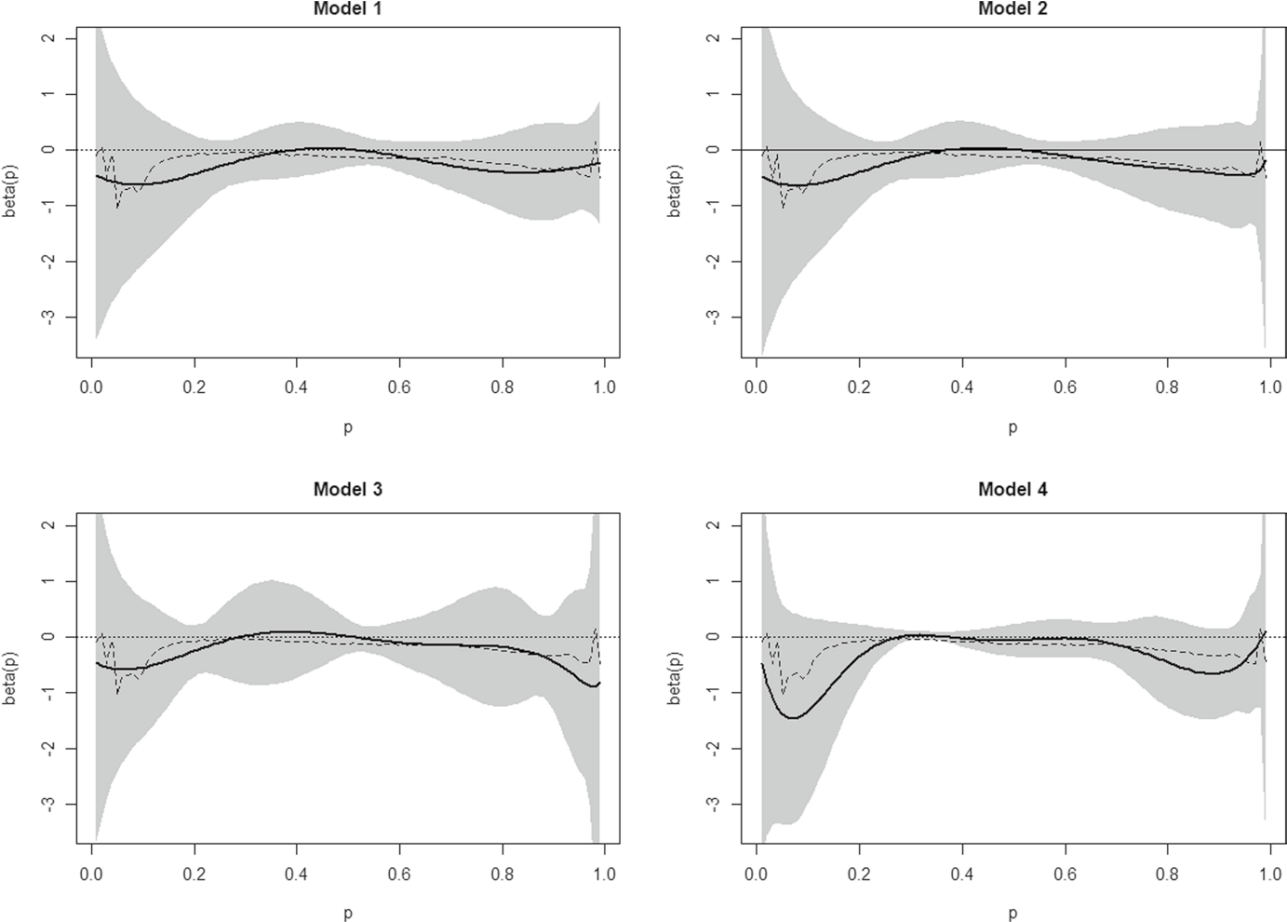
Estimates of $\beta _{2}(p\vert \varvec{\theta })$ under Models 1, 2, 3 and 4: QRCM estimates (*solid line*) with 95% confidence bands (*shaded area*) and jittering estimates at $p={0.01,0.02,...,0.99}$ (*broken line*). Degree program in Psychology

Selecting a QRCM model is not straightforward. In fact, a more complex model can reduce the integrated loss function, but it is more prone to overfitting, while it does not necessarily improve the inference on the coefficient of $X_2$ (cohort 2019), measuring the effect of remote teaching. Therefore, we compared the models on the basis of the plots of $\beta _{2}(p\vert \varvec{\theta })$: each panel of Fig. 2 displays the jittering estimates at the percentiles ($p={0.01,0.02,...,0.99}$) and the QRCM fitted quantile function with confidence bands.

We selected Model 2 since it achieves a satisfactory balance between parsimony and accurate interpolation of the non-parametric estimates obtained with jittering. We discard Models 3 and 4 despite the lower value of the loss function. In fact, Model 3 extends Model 2 with a finer specification for the control variables $X_1, X_3, X_4$, yielding a marked increase in the confidence bands. On the other hand, Model 4 extends Model 2 with a finer specification of the intercept and the coefficient of cohort $X_2$, with confidence bands decreasing for some values of *p* and increasing otherwise. It is worth to note that the additional parameters of Models 3 and 4 do not improve the interpolation of the jittering estimates for $X_2$, which slightly deteriorates instead. Therefore, we retain Model 2 even if it is not fully satisfactory in terms of global fit (Kolmogorov-Smirnov goodness-of-fit statistic$=0.0634$, bootstrap *p*-value$<0.001$). This case study illustrates the difficulties in model selection and the conflict between global fit and local fit.

It is reassuring to note that the choice of a specific model is essentially irrelevant for our research question on the effect of remote teaching: in fact, Fig. 2 shows that all models under consideration lead to the same finding, i.e., the effect of remote teaching is essentially null in the centre of the distribution and negative in the tails. However, the 95% confidence bands always overlap the zero, thus the estimated effect does not reach statistical significance.

An advantage of the QRCM approach is the availability of a global test on the effect of a covariate, in addition to quantile-specific tests. The global test is carried out as a Wald test for the null hypothesis that all coefficients associated to a covariate are zero. In our application, the cohort effect does not attain significance at a 5% level (*p*-value = 0.86).

**Fig. 3:**
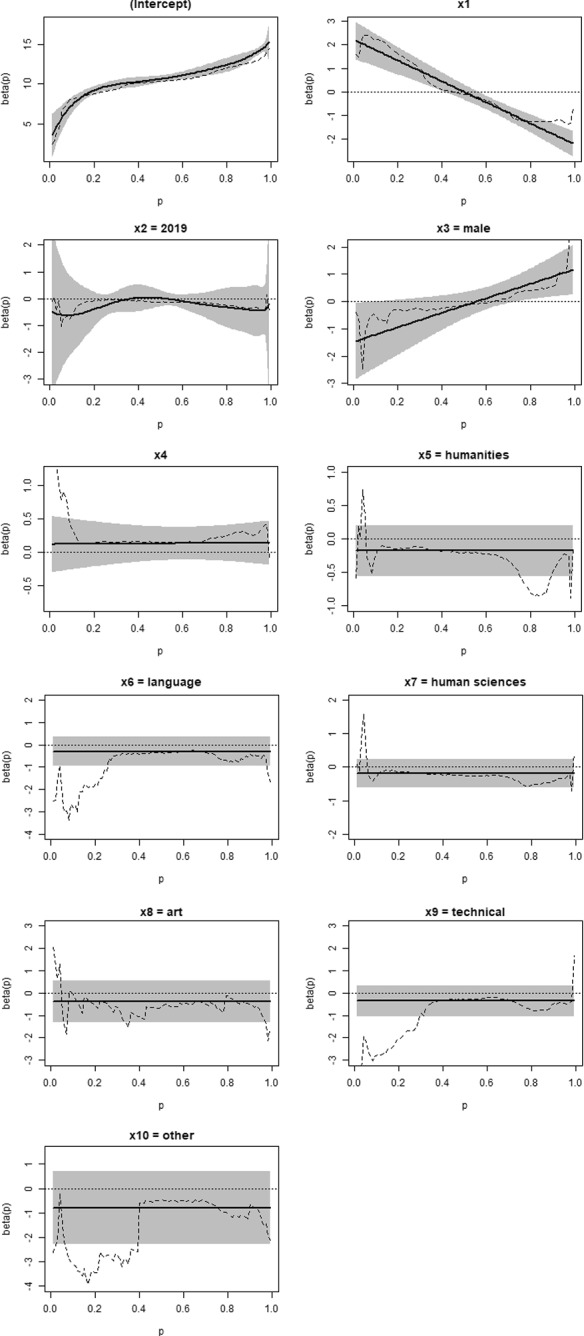
QRCM estimates of $\beta _{c}(p\vert \varvec{\theta })$ under Model 2 (*solid line*) with 95% confidence bands (*shaded area*) and jittering estimates at $p={0.01,0.02,...,0.99}$ (*broken line*). Degree program in Psychology

Figure 3 shows the quantile functions of the QRCM approach obtained from Model 2 of Table 2, alongside with 95% confidence bands. For comparison, broken dashed lines are plotted for jittering estimates at the percentiles ($p={0.01,0.02,...,0.99}$). Table 3 displays the estimates for the two approaches at five quantiles ($p={0.10, 0.25, 0.50, 0.75, 0.90}$), together with standard errors (those of jittering are computed with bootstrap using 100 iterations).

**Table 3:** Estimated quantile regression coefficients at selected quantile orders obtained with jittering and QRCM under Model 2 (standard errors in parenthesis). Degree program in Psychology

	jittering					QRCM				
	$p=0.10$	$p=0.25$	$p=0.50$	$p=0.75$	$p=0.90$	$p=0.10$	$p=0.25$	$p=0.50$	$p=0.75$	$p=0.90$
Intercept	8.101	9.050	10.369	11.608	12.643	7.374	9.668	10.623	12.049	13.482
	(0.362)	(0.271)	(0.096)	(0.305)	(0.224)	(0.505)	(0.260)	(0.224)	(0.418)	(0.322)
Credits 1*st* sem. (normalised)	2.253	1.323	−0.103	−1.212	−1.219	1.777	1.107	−0.008	−1.123	−1.793
	(0.313)	(0.290)	(0.081)	(0.216)	(0.186)	(0.354)	(0.272)	(0.164)	(0.171)	(0.232)
Cohort 2019	0.661	−0.058	−0.121	−0.199	−0.317	−0.621	−0.244	0.012	−0.287	−0.425
	(0.541)	(0.108)	(0.061)	(0.198)	(0.262)	(0.716)	(0.202)	(0.153)	(0.308)	(0.453)
Male	−0.531	−0.296	−0.143	0.362	0.561	−1.209	−0.810	−0.145	0.521	0.920
	(0.918)	(0.456)	(0.119)	(0.347)	(0.362)	(0.615)	(0.461)	(0.245)	(0.253)	(0.376)
High school grade (normalised)	0.345	0.164	0.152	0.216	0.276	0.122	0.126	0.131	0.137	0.141
	(0.252)	(0.074)	(0.042)	(0.105)	(0.127)	(0.192)	(0.162)	(0.128)	(0.132)	(0.152)
High School type (ref: scientific)										
Humanities	−0.220	−0.120	-0.220	−−0.540	−0.492	−0.177	−0.177	−0.177	−0.177	−0.177
	(0.676)	(0.126)	(0.082)	(0.362)	(0.459)	(0.193)	(0.193)	(0.193)	(0.193)	(0.193)
Language	−2.732	−1.177	−0.354	−0.503	−0.445	−0.297	−0.297	−0.297	−0.297	−0.297
	(1.382)	(0.883)	(0.151)	(0.397)	(0.411)	(0.327)	(0.327)	(0.327)	(0.327)	(0.327)
Human sciences	−0.248	−0.151	−0.251	−0.467	−0.417	−0.198	−0.198	−0.198	−0.198	−0.198
	(0.377)	(0.134)	(0.089)	(0.405)	(0.332)	(0.217)	(0.217)	(0.217)	(0.217)	(0.217)
Art	0.004	−0.477	−0.556	−0.649	−0.555	−0.396	−0.396	−0.396	−0.396	−0.396
	(1.713)	(1.106)	(0.563)	(0.785)	(0.634)	(0.475)	(0.475)	(0.475)	(0.475)	(0.475)
Technical	−2.792	−1.682	−0.292	−0.553	−0.506	−0.354	−0.354	−0.354	−0.354	−0.354
	(1.006)	(0.906)	(0.149)	(0.365)	(0.442)	(0.349)	(0.349)	(0.349)	(0.349)	(0.349)
Other	−3.158	−2.720	−0.483	−0.778	−0.672	−0.789	−0.789	−0.789	−0.789	−0.789
	(1.079)	(1.135)	(0.898)	(0.405)	(0.591)	(0.765)	(0.765)	(0.765)	(0.765)	(0.765)

The coefficients estimated with QRCM (parametric modelling of the coefficients) are close to those of the jittering approach, especially for the parameters with more flexible functions, i.e. the intercept and the coefficient of $X_2$ (cohort). The discrepancies are larger in the tails of the distribution of the response variable, where there is higher uncertainty and the smoothing operated by QRCM is stronger.

The standard errors are generally higher in the tails. Neither approach guarantees more precision for all variables at all considered quantiles. Specifically, QRCM provides smaller standard errors for most of the estimated coefficients, especially for the smallest and highest quantiles. However, QRCM yields larger standard errors for the dummy of cohort due to the flexible specification.

Table 4 reports the average standard errors at five quantiles. In both approaches the standard errors are larger in the tails. Interestingly, with jittering the standard errors are markedly larger on the left tail, but this asymmetry is much attenuated with QRCM. The ratios of the average standard errors show that the QRCM approach provides more precision at all considered quantiles except the median. The gain is higher in the left tail, where the standard errors of the jittering estimates are particularly large. Overall, these findings demonstrate that the smoothing operated by the QRCM approach leads to an increased precision in the tails of the distribution, where the local fitting of the jittering approach is worsening.

**Table 4:** Average standard errors at selected quantile orders obtained with jittering and QRCM under Model 2. Degree program in Psychology

	$p=0.10$	$p=0.25$	$p=0.50$	$p=0.75$	$p=0.90$
jittering	0.784	0.499	0.212	0.354	0.366
QRCM	0.428	0.335	0.295	0.328	0.310
Ratio	0.546	0.671	1.392	0.927	0.959

Appendix 1 reports the results of the analysis concerning the degree program in Industrial Design. For the QRCM approach we adopted the selection procedure outlined above: even if the selected model is different, the reflections made in this Section remain valid. The effect of remote teaching is positive, though not statistically significant. As compared with Psychology, the confidence bands of QRCM are larger because of the smaller sample size, but the gain in efficiency of QRCM over jittering is higher.

## Final remarks

Quantile regression is an appealing methodology for modelling a count variable with an irregular distribution, such as the number of credits earned by university students. Motivated by the aim of evaluating the impact of remote teaching on student productivity, we analysed data from the University of Florence using the traditional approach based on jittering (Machado and Santos Silva 2005) and the quantile regression coefficients modelling (QRCM) approach proposed by Frumento and Salvati (2021).

The QRCM approach provides estimates of the quantile regression coefficients that are smooth functions of the quantile order *p*. The smoothing is especially convenient in the tails of the distribution, preventing abrupt changes in the point estimates and increasing precision. We detected this pattern in the analysis of both degree programs. Another advantage of the coefficients modelling approach is the reduced computational time since optimising a single objective function yields estimates at all quantiles. On the contrary, traditional quantile regression entails fitting the model separately at every quantile of interest; moreover, to obtain average-jittering estimates, the jittering and estimation steps have to be repeated many times.

The main difficulty with the coefficients modelling approach lies in model selection. Indeed, in addition to the quantile regression function, one has to specify parametric functions for all regression coefficients; thus, the range of options is extremely wide and the risk of overfitting is serious. The jittering approach is pivotal as an exploratory tool to guide the choice of the parametric functions. In our case study, model selection was facilitated by the presence of a coefficient of primary interest, namely the coefficient of cohort 2019 measuring the effect of remote teaching. Our strategy was to adopt complex functions for the intercept, which represents the baseline pattern, and the coefficient for the effect of remote teaching. On the other hand, for the coefficients of the control variables, we adopted simple functions which were evaluated not only in terms of global fit but also by monitoring the changes in the inferences for the effect of remote teaching. Indeed, we detected a bias-variance trade-off since highly flexible specifications of the control variables were associated with a loss of precision in estimating the coefficient of primary interest.

A difficulty of the coefficients modelling approach is that model selection cannot rely on standard likelihood-based methods since estimation depends on an integrated loss function. The minimised value of the loss function can be used to choose among models with the same number of parameters. Still, there is no criterion to compare models of different complexity. Another tool to guide model selection is the goodness-of-fit test of Frumento and Bottai (2016), which was derived for continuous outcomes. Using this test with count data seems reasonable, but its behaviour has to be investigated.

The case study assessed the impact of remote teaching after COVID-19 on the productivity of university students. Controlling for background characteristics and productivity during the previous semester, we found that first-year students of Psychology at the University of Florence were little affected by the switch to remote teaching. Indeed, we detected minor negative effects in the tails without reaching statistical significance. On the other hand, the analysis with first-year students of Industrial Design revealed positive effects of remote teaching at all quantiles. However, the smaller sample size gives larger confidence bands; thus, the effects are not statistically significant. As for interpretation, note that we estimated an overall effect of the changes in didactic activities to contrast the COVID-19 pandemic: in fact, remote teaching also implied remote examinations, and our data do not allow us to disentangle the effect of teaching itself from the effect due to examinations.

As for the methodology, our case study confirm the merits of quantile regression coefficients modelling, especially for the smoothness of estimates in the tails of the distribution, alongside increased precision. Nonetheless, model selection is challenging; thus, we recommend using the traditional jittering approach as a reference. The spread of the coefficients modelling approach depends critically on developing effective tools for model selection.

The topic of flexible modelling of count data is rapidly evolving. Two recent proposals are noteworthy. Peluso et al. (2019) developed a generalised additive model with Discrete Weibull distribution. The proposed method is compared with jittering via simulation, finding a reduction in the RMSE (about $5\%$ for a sample size $n=1000$). Moreover, their application to the number of planned children highlights the greater stability of the inferential results in the tails. From a different perspective, Geraci and Farcomeni (2022) developed a two-step estimator for implementing quantile regression with the mid-quantile function. For discrete random variables, this amounts to smooth the quantile function. In a simulation study, this method returned estimates of the slopes similar to the jittering approach, with a gain in efficiency for central quantiles ($0.2<p<0.8$) and no gain (or loss for $n=100$) at $p=0.2, 0.8$. Future research should comprehensively compare quantile regression coefficients modelling with the alternative approaches.

## Figures and Tables

**Fig. 4:**
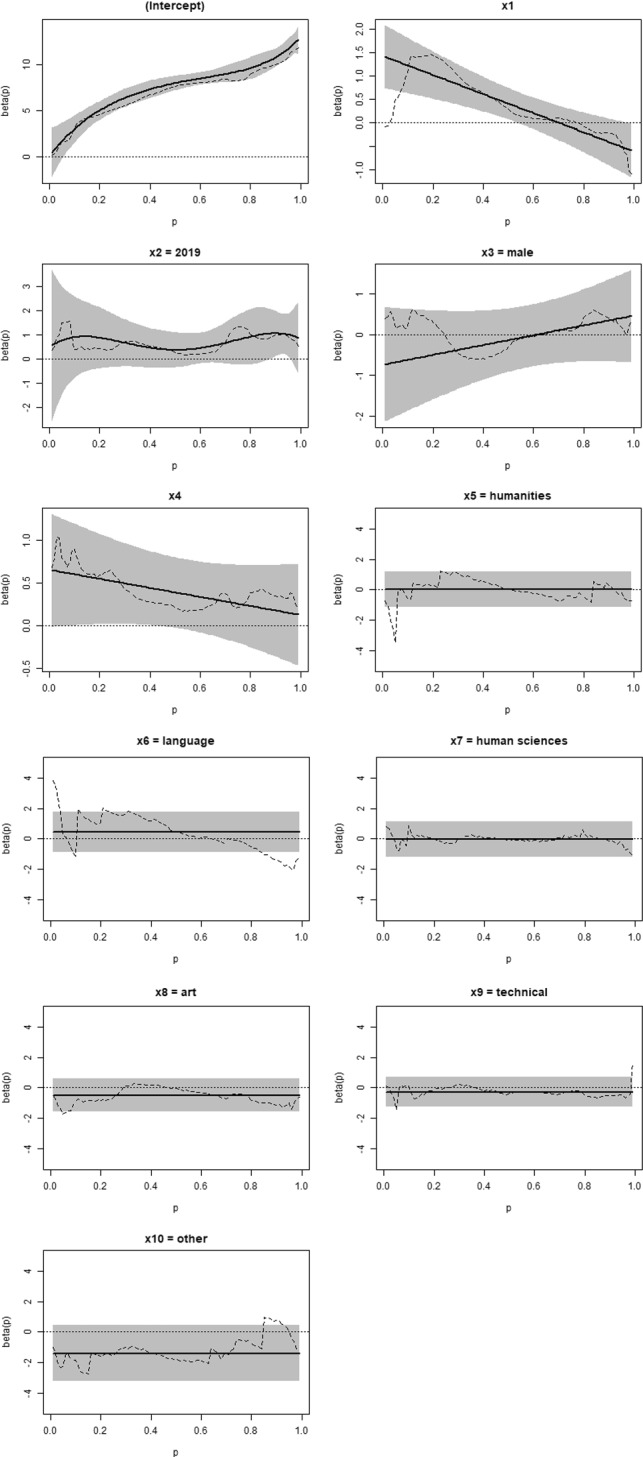
QRCM estimates of $\beta _{c}(p\vert \varvec{\theta })$ (*solid line*) with 95% confidence bands (*shaded area*) and jittering estimates at $p={0.01,0.02,...,0.99}$ (*broken line*). Degree program in Industrial Design

## Appendix A: Results for industrial design

We report the results of the analysis concerning the degree program in Industrial Design of the University of Florence. The strategy of model building is the same adopted for the degree program in Psychology (Sect. 3.3). The selected model for the QRCM approach has the following specification:

$$\begin{aligned}&\beta _{0}(p\vert \varvec{\theta })= \theta _{00} + \theta _{01}p + \theta _{02}p^2 + \theta _{03}p^3 + \theta _{04}p^4 -\theta _{05}\log (1-p)&\\&\beta _{2}(p\vert \varvec{\theta })= \theta _{20} + \theta _{21}p + \theta _{22}p^2 + \theta _{23}p^3 + \theta _{24}p^4 - \theta _{25}\log (1-p) \\&\beta _{c}(p\vert \varvec{\theta })= \theta _{c0} + \theta _{c1}p \quad \text {for} \, c \in \{1,3,4\}\\&\beta _{c}(p\vert \varvec{\theta })= \theta _{c0} \quad \text {for} \, c=5,6,\dotsc ,10 \end{aligned}$$

We imposed a structure similar to the other degree program: the intercept and the coefficient of primary interest are specified with flexible functions, whereas the other coefficients are approximated with a line or a constant. The main difference lies in the selected functional forms for $\beta _{0}(p\vert \varvec{\theta })$ and $\beta _{2}(p\vert \varvec{\theta })$.

Figure 4 shows the plots of the estimates obtained with jittering and QRCM, corresponding to Fig. 3. Table 5 displays the estimates obtained with jittering and QRCM at selected quantiles, corresponding to Table 3.

**Table 5:** Estimated quantile regression coefficients at selected quantile orders obtained with jittering and QRCM (standard errors in parenthesis). Degree program in Industrial Design

	jittering					QRCM				
	$p=0.10$	$p=0.25$	$p=0.50$	$p=0.75$	$p=0.90$	$p=0.10$	$p=0.25$	$p=0.50$	$p=0.75$	$p=0.90$
Intercept	3.022	5.109	7.672	8.302	10.061	3.046	5.792	8.017	9.291	10.824
	(1.010)	(0.604)	(0.432)	(0.522)	(0.415)	(0.744)	(0.501)	(0.404)	(0.549)	(0.500)
Credits $1{st}$ sem. (normalised)	1.084	1.300	0.279	0.034	−0.195	1.230	0.924	0.413	−0.098	−0.404
	(0.500)	(0.317)	(0.191)	(0.173)	(0.200)	(0.303)	(0.239)	(0.172)	(0.200)	(0.253)
Cohort 2019	0.392	0.423	0.279	1.320	0.981	0.904	0.784	0.372	0.795	1.058
	(1.165)	(0.538)	(0.364)	(0.521)	(0.417)	(0.864)	(0.573)	(0.373)	(0.527)	(0.481)
Male	0.178	0.001	−0.297	0.100	0.390	−0.618	−0.438	−0.137	0.164	0.344
	(0.828)	(0.713)	(0.481)	(0.552)	(0.726)	(0.634)	(0.518)	(0.395)	(0.426)	(0.512)
High School grade (normalised)	0.901	0.627	0.214	0.203	0.355	0.599	0.520	0.388	0.257	0.178
	(0.438)	(0.330)	(0.208)	(0.239)	(0.203)	(0.303)	(0.255)	(0.210)	(0.232)	(0.272)
High School type (ref: scientific)										
Humanities	−0.503	1.152	0.000	−0.381	0.283	0.004	0.004	0.004	0.004	0.004
	(2.534)	(1.315)	(0.483)	(1.027)	(1.020)	(0.593)	(0.593)	(0.593)	(0.593)	(0.593)
Language	1.492	1.668	0.406	−0.119	−1.436	0.430	0.430	0.430	0.430	0.430
	(2.466)	(1.773)	(0.751)	(0.653)	(0.686)	(0.685)	(0.685)	(0.685)	(0.685)	(0.685)
Human sciences	0.907	−0.291	−0.088	0.129	−0.139	−0.053	−0.053	−0.053	−0.053	−0.053
	(1.356)	(0.890)	(0.572)	(0.687)	(0.639)	(0.594)	(0.594)	(0.594)	(0.594)	(0.594)
Art	−0.846	−0.563	−0.069	−0.386	-1.156	-0.496	-0.496	-0.496	-0.496	−0.496
	(1.061)	(0.961)	(0.670)	(0.565)	(0.613)	(0.556)	(0.556)	(0.556)	(0.556)	(0.556)
Technical	0.155	0.016	−0.378	−0.192	−0.508	−0.291	−0.291	-0.291	−0.291	−0.291
	(1.149)	(0.730)	(0.521)	(0.779)	(0.723)	(0.499)	(0.499)	(0.499)	(0.499)	(0.499)
Other	−1.839	−1.536	−1.825	−0.511	0.766	−1.398	−1.398	−1.398	−1.398	−1.398
	(1.391)	(1.056)	(0.857)	(1.695)	(1.428)	(0.931)	(0.931)	(0.931)	(0.931)	(0.931)

Finally, Table 6 compares the average standard errors under the two approaches, similarly to Table 4. In this case the QRCM approach entails a gain in precision at any quantile order. The gain is pretty small at the median and it increases while moving towards more extreme quantiles, especially on the left.

**Table 6:** Average standard errors at selected quantile orders obtained with jittering and QRCM. Degree program in Industrial Design

	$p=0.10$	$p=0.25$	$p=0.50$	$p=0.75$	$p=0.90$
jittering	1.263	0.839	0.503	0.674	0.643
QRCM	0.610	0.540	0.492	0.527	0.534
Ratio	0.483	0.644	0.979	0.782	0.831
